# Draft Genome Sequence of the Fruiting Myxobacterium *Nannocystis exedens* DSM 71

**DOI:** 10.1128/genomeA.01227-17

**Published:** 2017-10-26

**Authors:** Anke Treuner-Lange, Marc Bruckskotten, Oliver Rupp, Alexander Goesmann, Lotte Søgaard-Andersen

**Affiliations:** a Department of Ecophysiology, Max Planck Institute for Terrestrial Microbiology, Marburg, Germany; b Bioinformatics and Systems Biology, Justus-Liebig University Giessen, Giessen, Germany

## Abstract

In response to starvation, members of the order *Myxococcales* form morphologically very different fruiting bodies. To determine whether fruiting myxobacteria share a common genetic program that leads to fruiting body formation, we sequenced and assembled the genome of *Nannocystis exedens* DSM 71 as two contigs with a total GC content of 72%.

## GENOME ANNOUNCEMENT

Most members of the order *Myxococcales* initiate a developmental program in response to starvation that results in the formation of a multicellular fruiting body, inside which the rod-shaped cells differentiate to spores (1, 2). Based on phylogenetic analyses using 16S rRNA sequences, a deep trifurcation of members of the order *Myxococcales* has repeatedly been observed (3–5). Accordingly, this order is divided into three suborders, i.e., *Cystobacterineae*, *Sorangineae*, and *Nannocystineae*. Currently, the order includes 28 genera and 55 species (6).

While fruiting body formation in the model organism *Myxococcus xanthus*, a member of the suborder *Cystobacterineae*, is relatively well understood (7, 8), much less is known about the genetic basis underlying fruiting body formation in the remaining suborders. Of the 20 complete (9–26) and 36 draft *Myxococcales* genomes (27–34), only 4 are from members of the suborder *Nannocystineae*. Members of this suborder form fruiting bodies that are either solitary or aggregated sporangioles. To generate an additional resource for accurate genome comparisons, we sequenced and annotated the complete genome of *Nannocystis exedens* strain DSM 71, which was obtained from the Deutsche Sammlung von Mikroorganismen und Zellkulturen GmbH.

After verifying the ability of *N. exedens* DSM 71 to form irregularly shaped sporangia containing myxospores as described previously (35), we collected genomic DNA (36) from liquid cultures and sequenced it using PacBio single-molecule real-time (SMRT) sequencing (37) on the PacBio RSII platform at the Max Planck-Genome-Centre, Cologne, Germany. Eight SMRT cells were used. Additionally, 11,834,547 100-bp paired-end Illumina reads were obtained using the HiSeq2000 platform. After quality evaluation and filtering of 282,467 PacBio subreads, assembly using the Hierarchical Genome Assembly Process (38) resulted in two contigs with a 94-fold coverage. These two contigs cover approximately 12.1 Mb (11.3 Mb and 0.8 Mb) with a similar GC content of 72%. Additionally, the Illumina reads were applied to correct the assembled contigs using the Pilon tool (39). Due to complex and large repetitive regions in at least two areas of the genome, as well as missing coverage in these regions, we were unable to fully close the genome. The genome annotation was prepared using Prokka (40). A total of 9,278 protein-coding sequences were identified, together with 107 tRNAs and 9 rRNA operons. BLASTp searches against the RefSeq database were used to assign functional annotation and identify possible frameshifts in genes. The corresponding genes were removed from the annotation.

Alignment of the *N. exedens* DSM 71 genome with other genomes from the order *Myxococcales* using NUCmer (41) revealed overall synteny to the *N. exedens* ATCC 25963 genome, with 97% of the sequences aligning. The remaining three *Nannocystineae* genomes did not match significantly.

The *N. exedens* DSM 71 genome sequence offers valuable data for studying the evolution of the genetic programs leading to fruiting body formation and also provides a resource for identifying the novel genetic determinants that are important for fruiting body formation and morphology.

### Accession number(s).

This whole-genome shotgun project has been deposited at DDBJ/ENA/GenBank under the accession number NETK00000000. The version described in this paper is the first version, NETK01000000.
